# Identifying overlapping mutated driver pathways by constructing gene networks in cancer

**DOI:** 10.1186/1471-2105-16-S5-S3

**Published:** 2015-03-18

**Authors:** Hao Wu, Lin Gao, Feng Li, Fei Song, Xiaofei Yang, Nikola Kasabov

**Affiliations:** 1 School of Computer Science and Technology, Xidian University, Xi'an, Shaanxi 710071, China; 2 Knowledge Engineering and Discovery Research Institute, Auckland University of Technology, Auckland, New Zealand

**Keywords:** Driver pathways, Network-based method, Somatic mutations, High coverage, High exclusivity

## Abstract

**Background:**

Large-scale cancer genomic projects are providing lots of data on genomic, epigenomic and gene expression aberrations in many cancer types. One key challenge is to detect functional driver pathways and to filter out nonfunctional passenger genes in cancer genomics. Vandin *et al*. introduced the Maximum Weight Sub-matrix Problem to find driver pathways and showed that it is an NP-hard problem.

**Methods:**

To find a better solution and solve the problem more efficiently, we present a network-based method (NBM) to detect overlapping driver pathways automatically. This algorithm can directly find driver pathways or gene sets *de novo *from somatic mutation data utilizing two combinatorial properties, high coverage and high exclusivity, without any prior information. We firstly construct gene networks based on the approximate exclusivity between each pair of genes using somatic mutation data from many cancer patients. Secondly, we present a new greedy strategy to add or remove genes for obtaining overlapping gene sets with driver mutations according to the properties of high exclusivity and high coverage.

**Results:**

To assess the efficiency of the proposed NBM, we apply the method on simulated data and compare results obtained from the NBM, RME, Dendrix and Multi-Dendrix. NBM obtains optimal results in less than nine seconds on a conventional computer and the time complexity is much less than the three other methods. To further verify the performance of NBM, we apply the method to analyze somatic mutation data from five real biological data sets such as the mutation profiles of 90 glioblastoma tumor samples and 163 lung carcinoma samples. NBM detects groups of genes which overlap with known pathways, including P53, RB and RTK/RAS/PI(3)K signaling pathways. New gene sets with p-value less than 1e-3 are found from the somatic mutation data.

**Conclusions:**

NBM can detect more biologically relevant gene sets. Results show that NBM outperforms other algorithms for detecting driver pathways or gene sets. Further research will be conducted with the use of novel machine learning techniques.

## Background

Cancer has become one of the most serious threats to human health. Cancer is driven in part by somatic mutations, including single nucleotide substitutions, small indels, large copy number aberrations, and structural aberrations that accumulate during the lifetime of an individual [2]. At the same time, a large number of somatic mutations have been discovered in cancer genomes [3]. One key challenge in interpreting these data is to distinguish functional driver mutations, which lead to tumorigenesis, from passenger mutations, which are functionally neutral and have no consequence for cancer [4]. The second key challenge is to detect biological driver pathways, which are frequently perturbed within some tumor cells, and cause the product of tumorigenic properties, such as cell angiogenesis, metastasis or proliferation.

The final decision of whether a gene mutation is a driver or a passenger is to be made after testing its biological function. However, it is expensive at present to detect somatic mutations and to validate their functions experimentally [5]. A common approach to detect driver mutations is to detect genes with recurrent mutations in a large number of cancer patients. The standard technology to detect recurrently mutated genes is to test a single gene whether its frequency of mutations is significantly higher than expected [1]. This statistical approach has been used to detect many important cancer genes, but it can't be used to identify driver mutation pathways and driver genes in cancer.

It is an urgent priority to predict driver mutated genes and pathways through computational approaches. A large number of computational methods have been developed to address these challenges. There are mainly two computational approaches: one approach is to infer driver mutated genes and pathways by integrating somatic mutation data and additional biological knowledge such as protein-protein interaction networks, gene expression data or other sources of information [2,4-6], and the other is to directly find driver pathways or groups of driver genes *de novo *from somatic mutation data utilizing two combinatorial properties, namely - coverage and exclusivity [1,7,8], rather than integrate any additional biological information and prior knowledge. These methods are successful in inferring the influence of some mutations, but generally do not result in overlapping driver pathways. Actually some genes may be involved in multiple biological processes, so these genes may be members of more than one driver pathway.

In recent years, the declining costs of genome sequencing allow measurement of the somatic mutations in many cancer genomes. Many research results, including those from The Cancer Genome Atlas (TCGA) website (https://tcga-data.nci.nih.gov/tcga/), Kyoto Encyclopedia of Genes and Genomes (KEGG) [9] and other projects, report a large number of significantly mutated genes. The analysis shows that driver mutations vary greatly between cancer patients, even for the same type of cancer [10]. One of the biological explanations for the mutated heterogeneity is that driver mutations not only target individual genes, but also target groups of genes in cellular regulatory and signaling pathways. Therefore, different cancer patients may hold different mutated gene members from a pathway of cancer development. Some methods have been introduced to look for enrichment in genes or groups of genes by prior biological knowledge, such as functional groups from GO [11] or known pathways from KEGG [9]. More recently, other methods have also been developed to detect recurrently mutated sub-networks by integrating somatic mutations and protein interaction networks.

The known gene and protein interaction networks in humans remain incomplete, and the information about the interactions in these networks is unreliable and imprecise [1,12]. The goal of identifying driver pathways is to reveal the natural data mutation properties and to gain some initial insights regarding mutated genes and pathways. Therefore, a good algorithm for finding mutated driver pathways should depend as little as possible on prior knowledge, which is usually not available before results analysis. Recently, the *De novo *Driver Exclusivity (Dendrix) [1] and Multi-Dendrix [7] algorithms have been introduced to detect driver pathways using combinatorial constraints derived from biological knowledge of how driver mutations emerge in pathways. Particularly, each cancer patient contains relatively few driver mutations, and these mutations perturb multiple cellular signaling or regulatory pathways. Each driver pathway contains approximately one driver mutation for one patient, so this is a pattern of mutual exclusivity between mutation genes in the driver pathway. Moreover, an important driver pathway should cover a large number of patients, so this is a pattern of high coverage by mutations. That is, a driver pathway corresponds to a set of genes that is mutated in a large number of patients, and whose mutations are mutually or approximately exclusive. These driver pathways of high coverage and mutually exclusivity are generally smaller and more focused than most signaling and regulatory pathways. The Multi-Dendrix algorithm [7] improves on Dendrix algorithm [1] in finding sets of genes with mutual exclusivity and high coverage. Dendrix calculates a single score for the weight of a set of genes, and finds the highest scoring set, then removes the set of genes found in previous iteration, and then repeats the step. Hence, the algorithm can discover multiple driver pathways iteratively. However, such an iterative approach can only yield local optimal sets of genes. Multi-Dendrix algorithm can identify multiple cancer driver pathways simultaneously by the Multiple Maximum Weight Sub-matrix problem using integer linear program [6].

To reduce the complexity of the solution and solve the NP-hard problem of the Maximum Weight Sub-matrix in an efficient approach, we present a new NBM algorithm to detect functional driver pathways *de novo *from somatic mutation data without any prior biological knowledge. In the first step, we filter the mutation matrix and reserve the genes which meet a certain frequency of recurrence, as genes altered in only one or few cancer patients may not be driver genes but passenger genes. So we remove the genes whose mutation numbers are lower than 5 percent of all cancer patients. In the second step, we construct a gene network by calculating the exclusive score between each pair of genes. If the exclusive score between a pair of genes is greater than or equal to a *threshold λ*, the edge between the pair will be created, and its weight is the exclusive score. In the third step, we present a novel greedy growth process based on the concept of high coverage and high exclusivity to find gene sets in the gene network constructed in the previous step. These gene sets are likely to correspond to driver pathways.

## Methods

### Exclusivity and coverage

**Figure 1:**
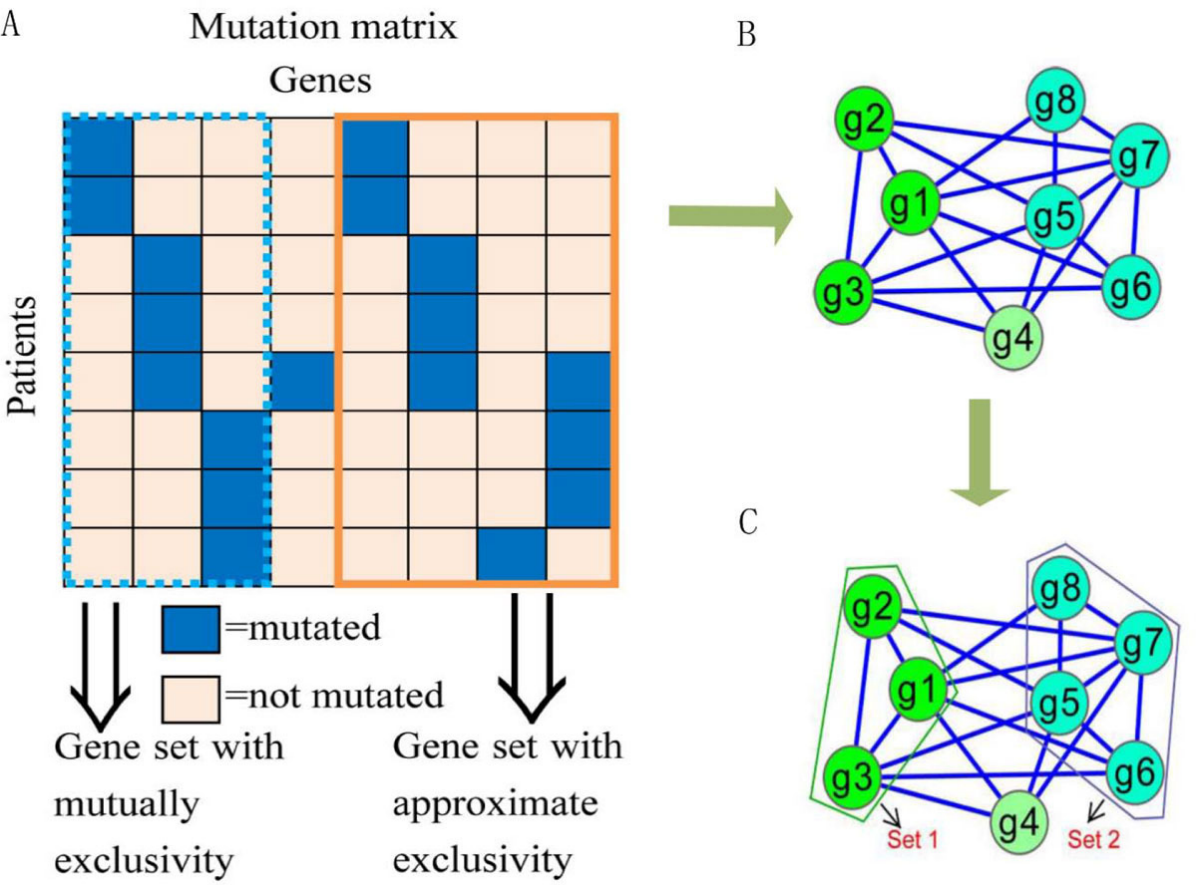
**The illustration process of the mutated driver pathways identification problem is shown in the figure**. A) Somatic mutation data in multiple patients are represented in a mutation matrix. B) Calculate the exclusive degree between each pair of genes, and construct a gene interaction network according to the exclusive degree. If the exclusive degree between the pair of genes is greater than or equal to *λ*, an edge is created between the two genes and the exclusive degree is represented as its weight. C) The exclusive degree of *Set 1 *is 1 and its coverage degree is also 1. If *g4 *is combined to *Set 1*, the exclusive degree of *Set 1 *will be reduced. The exclusive degree of *Set 2 *is 0.9 and the coverage degree of that is 1. If *g4 *is combined to *Set 2*, the exclusivity of *Set 2 *will be reduced.

Two important characteristics on the expected modes of somatic mutations have been used to understand the somatic mutation process of cancer in the last couple of years. Vandin *et al*. [1] introduced a measure to discover mutated driver pathways with two criteria from biological knowledge. The first one is "*high coverage*" which means many patients have at least one mutation in the pathway [6]; the second one is "*high exclusivity*" which means most patients have no more than one mutation in the pathway [6]. Given a binary mutation matrix *A *with *m *rows (samples) and *n *columns (genes), the *exclusive degree *function *ED*(*M*) and *coverage degree *function *CD*(*M*) are defined as follows. For a gene *g*, the *coverage *Γ(*g*) = {*i*: *A_ig _*- 1} represents the set of patients in which gene *g *is mutated (Figure 1). Similarly, for a sub-matrix *M *of size *m *× *k *in the mutation matrix *A*, the *coverage *is Γ(*M*) = ∪_*g*∈*M *_Γ(*g*). *M *is *mutually exclusive *if Γ(*g_j_*) ∩ Γ(*g_k_*) = ∅, for all *g_j_*, *g_k_*, ∈ *M*, *g_j _*≠ *g_k_*, 1 ≤ *j*, *k *≤ *n *A gene set in *A *named as a driver pathway is a column sub-matrix of *A *with *high coverage *and *high exclusivity*.

For a sub-matrix *M *of size *m *× *k*, the *exclusive degree *function *ED*(*M*) is defined as:

(1) $$ED(M)=\frac{\left|\Gamma (M)\right|}{\sum_{g\in M}\left|\Gamma (g)\right|}.$$

Obviously, for a pair of genes *g_j_*, *g_k_*, the *exclusive degree *of them is defined as:

(2) $$ED\left(g_{j},g_{k}\right)=\frac{\left|\Gamma \left(g_{j}\right)⋃\Gamma \left(g_{k}\right)\right|}{|\Gamma \left(g_{j}\right)|+\left|\Gamma \left(g_{k}\right)\right|}.$$

According to the above analysis, *ED*(*M*) = 1 when *M *is *mutually exclusive*.

For a sub-matrix *M *of size *m *× *k*, the *coverage degree *function *CD*(*M*) is defined as:

(3) $$CD\left(M\right)=\frac{\left|\Gamma \left(M\right)\right|}{m}.$$

Obviously, for a pair of genes *g_j_*, *g_k_*, the *coverage degree *of them is defined as:

(4) $$CD\left(g_{j},g_{k}\right)=\frac{\left|\Gamma \left(g_{j}\right)⋃\Gamma \left(g_{k}\right)\right|}{m}.$$

Note that *CD*(*M*) = 1 when *M *is the *complete coverage*.

The optimal results for a sub-matrix *M *are *ED*(*M*) = 1 and *CD*(*M*) = 1, but it is practically impossible. Obviously, we want to get the optimal results for the *exclusive degree *and the *coverage degree *at the same time, but increasing *coverage *may be obtained at the expense of decreasing *exclusivity*. So we define a function named *exclusivity-coverage-degree *as follows:

(5) $$ECD\left(M\right)=ED\left(M\right)\times CD\left(M\right)=\frac{{\left|\Gamma \left(M\right)\right|}^{2}}{m\times \sum_{g\in M}\left|\Gamma (g)\right|}.$$

Obviously, for a pair of genes *g_j_*, *g_k_*, the *exclusivity-coverage-degree *is defined as:

(6) $$ECD\left(g_{j},g_{k}\right)=ED\left(g_{j},g_{k}\right)\times CD\left(g_{j},g_{k}\right)=\frac{|\Gamma \left(g_{j}\right)⋃\Gamma \left(g_{k}\right){|}^{2}}{m\times \left(|\Gamma \left(g_{j}\right)|+\left|\Gamma \left(g_{k}\right)\right|\right)}.$$

Note that *ECD*(*M*) = 1 when *M *is the *mutually exclusive *and *complete coverage*. We need to find a gene set *M *whose *exclusive degree *and *coverage degree *are large simultaneously, so we define a function *ECD*(*M*) which allows for a tradeoff between *exclusivity *and *coverage*.

### Constructing a gene network based on approximate exclusivity

Vandin *et al*. introduced the Maximum Weight Sub-matrix Problem, which is defined for an integer *k *> 0 as the problem of looking for a *m *× *k *sub-matrix *M *of *A *that maximizes a weight *W*(*M*) [1,7,8], and showed that this problem is an NP-hard problem. To solve it more efficiently, we construct a weighted gene network based on *approximate exclusivity *between each pair of genes to simplify the relationships among genes and to reduce the calculation complexity greatly. First, we calculate the *exclusive degree *of each pair of genes in a somatic mutation matrix using formula (2). Second, we construct a gene network in which each node represents a gene and the weight of an edge represents the *exclusive degree *between the pair of genes that is greater than or equal to a *threshold λ*. If the *exclusive degrees *between one gene and the rest of all other genes are less than the *threshold λ*, the gene will not appear in the network. The process is shown in Figure 1.

### Clustering method in a gene network

Our clustering method consists of two steps. In the first step, the algorithm grows gene sets with *high coverage *and *high exclusivity *from selected two seeds. Initially, it selects two connected nodes with the highest *exclusivity-coverage-degree *as the first two seeds, and then grows the gene set from them using a greedy procedure. Whenever the growth process finishes, the algorithm selects the next two connected seeds by considering all the nodes that have not been included in any of gene sets found so far and taking the two connected seeds with the highest *exclusivity-coverage-degree *again. The whole procedure terminates when there are no two connected nodes remaining to consider.

A step-by-step description of the greedy growth process beginning from two connected nodes *v*_0_, *v*_1 _is as follows [13].

Step 1: Let *V*_0 _= {*v*_0_, *v*_1_}. Set the step number *t *= 0.

Step 2: Calculate the *exclusive degree *and *exclusivity-coverage-degree *of *V_t _*and let *V*_*t*+1 _= *V_t_*.

Step 3: For every external node *v *incident on at least one boundary edge, calculate the *exclusive degree *and *exclusivity-coverage-degree *of *V' *= *V_t _*∪ {*v*}. If *ECD*(*V'*) >*ECD*(*V*_*t*+1_) and *ED*(*V'*) >*λ*, let *V*_*t*+1 _= *V'*.

Step 4: For every internal node *v *which belongs to *V_t_*, calculate the *exclusive degree *and *exclusivity-coverage-degree *of *V' *= *V*_*t*_\{*v*}. If *ECD*(*V"*) >*ECD*(*V*_*t*+1_) and *ED*(*V"*) >*λ*, let *V*_*t*+1 _= *V"*.

Step 5: If *V*_*t *_≠ *V*_*t*+1_, increase *t *and return to step 2. Otherwise, declare *V*_*t *_a locally optimal driver pathway.

The growth process permits the removal of any node from the gene set being grown, including the original seed nodes. If the original one or two seed nodes are not included in the final driver pathway, they are considered outliers and they will not be included in any of the driver pathways, except in the case when another *high exclusivity *and *high coverage *cluster grown from different seed nodes absorbs them.

In the second step, we discard driver pathway candidates that contain less than three genes or those whose *coverage degree *is below a given *threshold δ*. For the gene sets whose *coverage degree *is too low, they are usually not regarded as driver pathways.

### Parameter settings

In the algorithm, *threshold λ *is used to decide whether there is an edge between each pair of genes according to its *exclusive degree*. *Threshold δ *is applied to determine whether a driver pathway candidate is chosen as a driver pathway according to its *coverage degree*. Based on the analysis of the algorithm and a large number of experiments, the following threshold values have been identified as leading to good results *λ *= 0.95 and *δ *= 0.3.

## Results and discussion

To assess the efficiency of NBM, we apply the method on simulated data and compare the results with Multi-Dendrix [7], with the iterative versions of Dendrix [1] and the RME methods [8]. When running all methods on a conventional computer, the NBM can obtain optimal results in less than nine seconds on a data set containing five pathways. Multi-Dendrix can obtain the results within a similar time to NBM, while Iterative-Dendrix and Iterative-RME obtain the results in more than 30 s. To assess the performance of NBM, we apply it onto five biological datasets (Table 1). Firstly, we remove the genes whose mutation frequency is lower than 5 percent of samples. Secondly, we construct a weighted gene network based on *approximate exclusivity *between each pair of genes from somatic mutation data. Finally, we obtain driver pathways based on the *exclusive degree *and *exclusivity-coverage-degree *using a new greedy strategy.

**Table 1:** The runtimes of the four algorithms on the same simulated dataset.

	Average runtime (seconds)			
*q*	Iterative-RME	Iterative-Dendrix	Multi-Dendrix	NBM
0.0001	30.26	635.68	5.32	7.25
0.0005	42.37	685.35	7.03	7.56
0.001	165.28	645.87	8.35	7.89
0.005	N/A	721.32	9.56	7.86
0.01	N/A	756.48	11.48	8.35
0.015	N/A	786.69	13.98	8.43
0.02	N/A	843.53	15.45	8.58

A comparison of the runtimes of Iterative-RME, Iterative-Dendrix, Multi-Dendrix and NBM on simulated mutation data with different passenger mutation probability *q*. Runtimes of each algorithm are shown as the mean of 20 runs for each *q*. N/A represents that Iterative-RME algorithm does not give any result within 24 hours. Simulations are performed on the machine running 64-bit windows with Intel Xeon 2.0 GHz processor and a maximum of 6 GB of available memory.

### Simulated data

We generate mutation data for *m *= 300 patients and *n *= 500 genes as follows. Five pathways *P *= (*P*_1_, *P*_2_, *P*_3_, *P*_4_, *P*_5_) with each *P*_*i *_containing five genes are implanted in the mutation matrix. We select the *coverage *Γ(*P_i_*) uniformly whose *coverage degrees CD*(*P_i_*) are 0.95, 0.85, 0.75, 0.65, 0.55, respectively. For each pathway *P_i_*, |Γ(*P_i_*)| patients are selected at random and a driver mutation is added to exactly one gene from the pathway *P_i_*. Therefore, in each pathway *P_i_*, the driver mutations are *mutually exclusive*. Then passenger mutations are added at random with probability of *q*.

We compare NBM with Multi-Dendrix [7], iterative versions of Dendrix [1] and RME [8] on the simulated mutation data. We calculate the average runtime of each algorithm on the simulated data. The runtimes of each algorithm are shown in Table 1 reflecting on different value of *q*.

### Biological data

To assess the performance of our NBM method on real biological data, we collect five somatic mutation data sets which are obtained from [2] directly. In Table 2 we present the information about the data sets, including number of patients, number of genes, maximum mutation frequency for all genes, average mutation number of each sample and average of mutation frequency for all genes.

**Table 2:** Basic information of the biological datasets used in this study.

Cancer type	#Patient	#Gene	MMF	AMN	AMF	NG
HNSCC [6]	74	4920	46	94.5	1.42	2
GBM1 [6]	84	178	43	9.6	4.5	8
GBM2 [2]	90	1126	48	21.8	1.74	5
LC [1]	163	356	64	6.0	2.75	4
OC [2]	313	5385	251	49.0	2.85	101

MMF: maximum mutation frequency for all genes. AMN: average mutation number of each sample. AMF: average of mutation frequency for all genes. NG: number of genes which are mutated in more than 20 samples in the mutation matrix. HNSCC: head and neck squamous cell carcinoma data. GBM1: glioblastoma multiforme data 1. GBM2: glioblastoma multiforme data 2. LC: lung carcinoma data. OC: ovarian carcinoma data.

We first apply NBM to the lung adenocarcinoma data set used by Vandin *et al*. [1] to assess its performance compared with Multi-Dendrix, Dendrix and RME. The NBM can obtain the driver pathways in less than 1 s, and Multi-Dendrix can get them in about 2 s, while Dendrix and RME can get them in more than 10 s. The analysis shows that the NBM is more efficient than the three other methods. We find that the four methods obtain either the same results or similar results. For instance, all the four methods can obtain the same gene set (TP53, ATM). RME, Dendrix and Multi-Dendrix can obtain gene set (KRAS, EGFR, STK11) with *k *= 3, and NBM can obtain also the gene set (KRAS, EGFR, NF1). The *exclusive degree *and *coverage degree *of the gene set (KRAS, EGFR, STK11) are 88.7% and 65.4% respectively, and the *exclusive degree *and *coverage degree *of the gene set (KRAS, EGFR, NF1) are 95.2% and 60.1% respectively. Obviously, the *exclusive degree *of our results is higher than that of the results obtained from the above three methods. It is necessary to acknowledge that the *coverage degree *declines at the expense of increasing *exclusive degree*. Besides, in our results, EGFR and NF1 are members of the RTK/RAS/PI(3)K signaling pathway involved in cellular proliferation, and EGFR and NF1 are associated with classical and mesenchymal subtypes, respectively. EGFR interacts with KRAS, and NF1 inhibits KRAS in the RTK signaling pathway [7]. Whereas the pair (KRAS, STK11) is not reported as significant using statistical tests [1]. Therefore, it is more reasonable for the gene set (KRAS, EGFR, NF1) to be a driver pathway.

In the following sections, we further apply our method NBM on the five datasets that are presented in Table 2. NBM can efficiently obtain the more biologically meaningful results in less time on all five data sets.

### Head and neck squamous cell carcinoma data (HNSCC)

HNSCC is a common and frequent lethal malignant tumor which is the sixth leading cancer according to incidence worldwide [6]. To uncover its mutational spectra, Stransky *et al *analyzed whole-exome sequencing data from 74 cancer-normal pairs and revealed many genes that have not been related to malignancy in previous studies [6]. The mutation matrix is sparse and only two genes are mutated in more than 20 samples. Six genes (TP53, TTN, SYNE1, MUC16, CSMD3, USH2A) are mutated in more than 10 samples. They are mutated in 46, 23, 15, 14, 12 and 11 samples respectively. We obtain three optimal gene sets shown in Table 3.

**Table 3:** Results of the algorithm in HNSCC.

Optimal gene sets	Gene number	Exclusivity	Coverage	P-value
TP53, DCHS1, PIK3CA	3	94.8%	74.3%	9.91e-04
SYNE1, CDKN2A, PCLO	3	97.0%	43.3%	4.95e-03
CSMD3, FAT1, TP63	3	96.2%	33.8%	7.49e-03

In the first driver pathway, TP53 is the core member of the p53 signaling pathway. DCHS1 can lead to a recessive syndrome in humans which includes periventricular neuronal heterotopias. These will affect the transcriptional effectors of the hippo signaling pathway [14]. PIK3CA is the core member of the RTK/RAS/PI(3)K signaling pathway involved in cellular proliferation [7]. TP53 binds directly to the PIK3CA promoter and inhibits its activity, and inactivation of TP53 and subsequent up-regulation of PIK3CA contribute to the pathophysiology of many human cancers. The gene set is altered in 74.3% with p-value = 9.91e-04. In the second driver pathway, SYNE1 has recently been identified in non-Hodgkin's lymphoma, renal cell carcinoma and all kinds of human cancers. Therefore, we have reason to believe that SYNE1 is the one needing more attention [15]. CDKN2A is the core member of the RB signaling pathway that is involved in G1/S progression. PCLO can target tumors using synthetic approaches by detecting tumor dependency on the inhibition of differentiation pathways, so it may become a therapeutic strategy in HNSCC. The detected gene set agrees with the optimal result of [2] when parameter *k *is 3. The gene set is altered in 43.3% with p-value = 4.95e-03. In the third driver pathway, CSMD3 mutation has been reported as an important factor in HNSCC [15]. FAT1 is usually thought to be a tumor suppressor, and loss of FAT1 may be predicted to allow loosening of the adhesions that usually restrain growth of migration of cells. The over-expression and genomic amplification of the TP63 locus can be observed in the majority of invasive HNSCCs [15]. The gene set is altered in 33.8% with p-value = 7.49e-03.

### Glioblastoma multiforme data 1 (GBM1)

Glioblastoma multiforme (GBM) is the most common and most aggressive type of primary brain tumor. In the GBM analysis, both copy-number aberrations and single-nucleotide (or small indel) mutations are contained to form mutation matrix [6]. Eight genes (CDKN2B, EGFR, CDKN2A, MTAP, PTEN, SEC61G, TP53 and ELAVL2) are mutated in more than 20 samples in the mutation matrix. They are mutated in 43, 38, 37, 34, 30, 28, 28 and 21 samples respectively. We get five optimal gene sets shown in Table 4.

**Table 4:** Results of the algorithm in GBM1.

Optimal gene sets	Gene number	Exclusivity	Coverage	P-value
CDKN2B, CYP27B1, RB1, ERBB2	4	90.9%	83.3%	6.46e-03
CDKN2A, TP53	2	90.8%	70.2%	5.33e-04
EGFR, NF1, KIT	3	93.4%	67.9%	4.99e-03
CDK4, MTAP, RB1, ERBB2	4	92.5%	73.8%	2.16e-05
PIK3CA, PTEN	2	90.4%	56.0%	9.91e-04
MDM2, NF1, PIK3R1	3	90.9%	47.6%	7.90e-03
DPYSL4, PDGFRA, PTEN, ERBB2	4	92.2%	56.0%	5.88e-05

In the first driver pathway, CDKN2B and RB1 are the core members of the cell cycle and cell cycle mitotic. CYP27B1 is the member of the glioblastoma copy number up and ERBB2 is the member of the cancer copy number up. The three genes (CDKN2B, CYP27B1, RB1) are sampled as the most frequent result in [1] when *k *is 3. The gene set is altered in 83.3% with p-value = 6.46e-03. In the second driver pathway, CDKN2A and TP53 are the members of the p53 signaling pathway. Somatic genetic CDKN2A and TP53 alterations are common in many human cancers and their precursors [16]. The gene set is altered in 70.2% with p-value = 5.33e-04. In the third driver pathway, EGFR and NF1 are the core members of the MAPK signaling pathway and KIT is the member of the cancer copy number up and the pathways in cancer. EGFR expression is associated with the development of the Schwann cell-derived tumors characteristic of NF1 [17]. The gene set is altered in 67.9% with p-value = 4.99e-03. In the fourth driver pathway, CDK4 and RB1 are the core members of the p53 signaling pathway. MTAP is the member of the WNT pathway and ERBB2 is the member of the ERBB signaling pathway. A recent study of CDK4 inhibitors in glioblastoma identified retinoblastoma tumor suppressor protein RB1 status as a determinant of tumor therapeutic efficacy [18]. The gene set is altered in 73.8% with p-value = 2.16e-05. In the fifth driver pathway, PIK3CA and PTEN are the core members of the RTK/RAS/PI(3)K signaling pathway which is prominently altered in glioblastoma, and PTEN inhibits PIK3CA in the RTK/RAS/PI(3)K signaling pathway. The gene set is altered in 56.0% with p-value = 9.91e-04. In the sixth driver pathway, MDM2 is the member of the p53 signaling pathway. NF1 is the core member of the MAPK signaling pathway and it usually affects RTK/RAS/PI(3)K signaling pathway. PIK3R1 is the member of the RTK/RAS/PI(3)K signaling pathway involved in cellular proliferation. The gene set is altered in 47.6% with p-value = 7.90e-03. In the seventh driver pathway, DPYSL4 usually targets genes TP53 and TP63. PDGFRA is the member of the MAPK signaling pathway. PTEN is the core member of the p53 and RTK/RAS/PI(3)K signaling pathways which are prominently altered in glioblastoma. ERBB2 is the member of the ERBB signaling pathway. The gene set is altered in 56.0% with p-value = 5.88e-05.

### Glioblastoma multiforme data 2 (GBM2)

The glioblastoma dataset is obtained from TCGA (2008). It contains gene expression profiles nucleotide sequence aberrations and DNA copy number alteration in 206 glioblastomas samples [6,19]. A mutation matrix covering 90 samples and 1126 genes are built [2]. Five genes (CDKN2B, CDKN2A, PTEN, MTAP and TP53) are mutated in more than 20 samples in the mutation matrix. They are mutated in 47, 44, 38, 34 and 25 samples respectively. We get seven optimal gene sets shown in Table 5.

**Table 5:** Results of the algorithm in GBM2.

Optimal gene sets	Gene number	Exclusivity	Coverage	P-value
CDKN2A, TP53, CDK4, RB1	4	82.4%	83.3%	2.64e-08
PTEN, PIK3CA, EGFR	3	86.7%	57.8%	1.17e-05
CDK4, CDKN2B, RB1	3	95.7%	73.3%	7.62e-05
PIK3CA, PTEN, PIK3R1	3	96.0%	53.3%	1.17e-05
MDM4, QKI, TP53, MDM2	4	93.2%	45.6%	2.74e-05
MDM2, TP53, PIK3R1, CPT1B	4	90.9%	44.4%	1.08e-04
CYP27B1, EGFR, NF1, PIK3R1	4	90.2%	51.1%	5.32e-04

**Figure 2:**
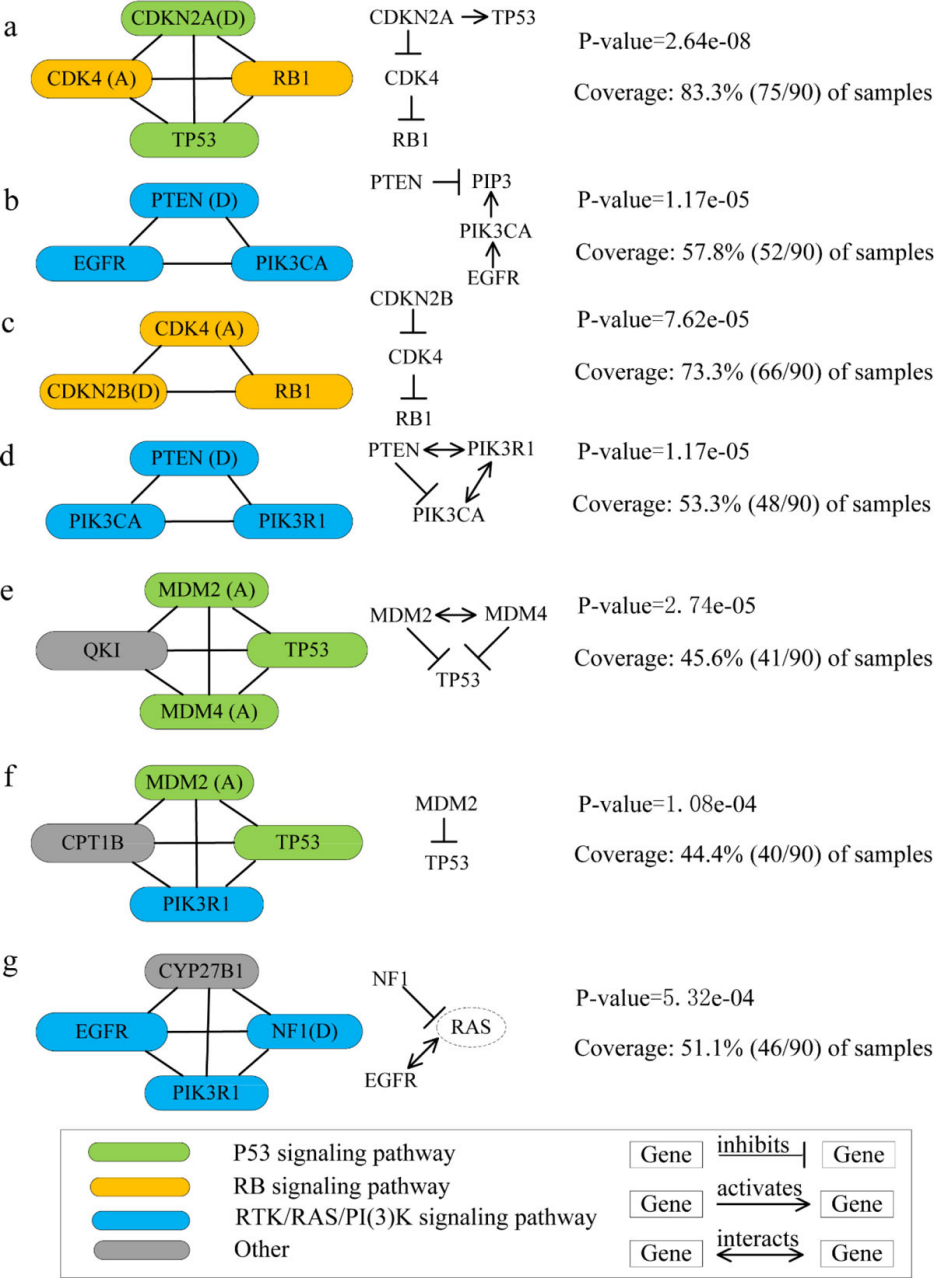
**The relationship between the genes in each module from the GBM dataset**. (Left) Nodes represent genes in seven modules found by NBM from GBM dataset. Genes with "(A)" appended are amplification mutations, genes with "(D)" appended are deletion mutations, and genes with no annotation are SNVs. Edges connect genes which appear in the same gene set. Color of nodes indicates membership in different signaling pathways as important for GBM: p53, RB and RTK/RAS/PI(3)K signaling pathways. (Right) Known interactions between genes in each driver pathway: inhibit, activate and interact. And p-value of each driver pathway is given from DAVID functional annotation tool whose website is http://david.abcc.ncifcrf.gov/summary.jsp.

In the first driver pathway, CDKN2A and TP53 are the core members of the p53 signaling pathway; CDKN2A, CDK4 and RB1 are the core members of the RB signaling pathway. CDKN2A activates TP53 in the p53 signaling pathway. CDKN2A inhibits CDK4 and CDK4 inhibits RB1 in the RB signaling pathway. The gene set is altered in 83.3% with p-value = 2.64e-8. In the second driver pathway, PTEN, PIK3CA and EGFR are the core members of the RTK/RAS/PI(3)K signaling pathway which is prominently altered in glioblastoma. PTEN inhibits PIP3; EGFR activates PIK3CA; PIK3CA activates PIP3 in the RTK/RAS/PI(3)K signaling pathway. The gene set is altered in 57.8% with p-value = 1.17e-05. In the third driver pathway, CDK4, CDKN2B and RB1 are the core members of the RB signaling pathway. CDK4 inhibits RB1, and CDKN2B inhibits CDK4 in the RB signaling pathway. The gene set is altered in 73.3% with p-value = 7.62e-05. In the fourth driver pathway, PIK3CA, PTEN and PIK3R1 are the core members of the RTK/RAS/PI(3)K signaling pathway which is prominently altered in glioblastoma. PTEN interacts with PIK3R1; PIK3R1 interacts with PIK3CA; PTEN inhibits PIK3CA in the RTK/RAS/PI(3)K signaling pathway. The gene set is altered in 53.3% with p-value = 1.17e-05. In the fifth driver pathway, MDM4, TP53 and MDM2 are the core members of the p53 signaling pathway. QKI is a member of the signal transduction and activation of RNA family of RNA-binding proteins, as a novel glioblastoma multiforme tumor suppressor [20]. The TP53 directly regulates QKI gene expression. MDM2 interacts with MDM4; MDM2 and MDM4 inhibit TP53; TP53 activates MDM2 in the p53 signaling pathway. The gene set is altered in 45.6% with p-value = 2.74e-05. In the sixth driver pathway, TP53 and MDM2 are the core members of the p53 signaling pathway. PIK3R1 is the core member of the RTK/RAS/PI(3)K signaling pathway which is prominently altered in glioblastoma. CPT1B is the member of the PPAR signaling pathway. MDM2 inhibits TP53 in the p53 signaling pathway. The gene set is altered in 44.4% with p-value = 1.08e-04. In the seventh driver pathway, PIK3R1, EGFR and NF1 are the members of the RTK/RAS/PI(3)K signaling pathway involved in cellular proliferation. NF1 usually affects RTK/RAS/PI(3)K signaling pathway. The mutational profile of CYP27B1 is nearly the same to a metagene and CYP27B1 is mutated in all patients as the metagene. EGFR and NF1 are associated with the classical and mesenchymal subtypes, respectively. Therefore, the exclusivity of mutations in the gene set is likely due to subtype-specific mutations [7]. EGFR interacts with RAS, and NF1 inhibits RAS in the RTK signaling pathway. The gene set is altered in 51.1% with p-value = 5.32e-04. We summarize the relationship of the genes in each module (Figure 2).

### Lung carcinoma data (LC)

Vandin *et al*. analyzed a collection of 1013 somatic mutations from 188 lung adenocarcinoma patients [21]. There are in total 356 genes reported to be mutated in at least one patient [1]. Four genes (TP53, KRAS, STK11 and EGFR) are mutated in more than 20 samples in the mutation matrix. They are mutated in 64, 60, 34 and 30 samples respectively. We get four optimal gene sets shown in Table 6.

**Table 6:** Results of the algorithm in LC.

Optimal gene sets	Gene number	Exclusivity	Coverage	P-value
TP53, ATM	2	98.7%	46.6%	1.74e-03
TP53, ATM, RB1	3	95.7%	48.6%	1.75e-04
APC, CDKN2A, EGFR, NF1	4	90.5%	35.0%	6.46e-03
KRAS, EGFR, NF1	3	95.2%	60.1%	2.99e-02

In the first driver pathway, TP53 and ATM are the core members of the p53 signaling pathway. ATM activates CHK1/2, and CHK1/2 activates TP53 in the p53 signaling pathway. The gene set is altered in 46.6% with p-value = 1.74e-3. In the second driver pathway, TP53, ATM and RB1 are the core members of the p53 signaling pathway. ATM has been reported as a mutated tumor suppressor gene in lung adenocarcinoma and it encodes a cell-cycle checkpoint kinase as a regulator of the p53 signal pathway [22]. ATM activates CHK1/2; CHK1/2 activates TP53; MDM2 inhibits RB1 and TP53 in the p53 signaling pathway. The gene set is altered in 48.6% with p-value = 1.75e-04. In the third driver pathway, APC is the member of the WNT signaling pathway, and CDKN2A is the member of the p53 signaling pathway. EGFR and NF1 are the core members of the MAPK signaling pathway and NF1 usually affects RTK/RAS/PI(3)K signaling pathway. EGFR inactivates RAS, and NF1 inhibits RAS in the MAPK signaling pathway. The gene set is altered in 35.0% with p-value = 6.46e-03. In the fourth driver pathway, KRAS is the core member of the MAPK signaling pathway which regulates cell differentiation and proliferation. EGFR is the core member of the RTK/RAS/PI(3)K signaling pathway. The two genes KRAS and EGFR are involved in the regulation of the mTOR pathway, whose dysregulation has been reported as important factor in lung adenocarcinoma [23]. NF1 is the most prominent case for a tumor suppressor, and its inactivating mutations can be found in neurofibromatosis type I patients [23]. The gene set is altered in 60.1% with p-value = 2.99e-02.

### Ovarian carcinoma data (OC)

Ovarian cancer often goes undetected until it has spread within the abdomen and pelvis. At this late stage, it is very difficult for ovarian cancer to treat and is frequently fatal [24]. The dataset is obtained from TCGA (2011) which has analyzed mRNA expression, microRNA expression, DNA copy number alteration and promoter methylation in 489 high-grade serous ovarian adenocarcinomas [2]. The mutation matrix is dense and 101 genes are mutated in more than 20 samples in the mutation matrix. Five genes (TP53, MYC, TTN, CCNE1, PPP2R2A) are mutated in more than 40 samples in the mutation matrix. They are mutated in 251, 81, 63, 54 and 42 samples respectively. We get four optimal gene sets shown in Table 7.

**Table 7:** Results of the algorithm in OC.

Optimal gene sets	Gene number	Exclusivity	Coverage	P-value
CCND2, TP53	2	94.5%	82.4%	1.74e-03
TLR3, TP53	2	94.8%	81.5%	8.26e-03
CCNE1, MYC, NINJ2	3	92.8%	49.2%	3.42e-03
CDH1, MAP3K1, TTN, MAP3K10	4	91.8%	35.8%	3.57e-03

In the first driver pathway, TP53 and CCND2 are the core members of the p53 signaling pathway. TP53 activates CCND2 in the p53 signaling pathway. The gene set is altered in 82.4% with p-value = 1.74e-03. In the second driver pathway, TLR3 is the member of the TLR signaling pathway which has been implicated as having both tumor-promoting and tumor-suppressive on ovarian cancer [25]. TP53 is the core member of the p53 signaling pathway. The gene set is altered in 81.5% with p-value = 8.26e-03. In the third driver pathway, the gene set identified by NBM is the same results as that given by BLP [2]. CCNE1 and MYC are involved in cell cycle and are two important genes engaged in cell cycle progression [26]. The gene set is altered in 49.2% with p-value = 3.42e-03. In the fourth driver pathway, CDH1 is the member of the pathway in cancer. MAP3K1 and MAP3K10 are the core members of the MAPK signaling pathway. TTN can increase the growth inhibition in ovarian cancer cells [27]. The gene set is altered in 35.8% with p-value = 3.57e-03.

## Conclusions

Finding mutated driver pathways in cancer is an essential problem in computational biology. In this paper, we introduce a novel algorithm called NBM for automatically discovering mutated driver pathways in cancer using somatic mutation data from many cancer patients. Our algorithm can find gene sets few mutated together in the same patient (high exclusivity) and mutated in many samples (high coverage). Notably, we find these mutation pathways *de novo *from the somatic mutation data of cancer without any prior biological knowledge of gene expression data, pathways and interactions between genes. In this algorithm, gene network is firstly constructed according to high exclusivity to solve the problem of high complexity encountered in the previous methods. Then a new greedy algorithm is introduced to cluster gene sets according to the properties of high exclusivity and high coverage. The results indicate that integrative analyses of somatic mutation data have the potential to detect gene sets pertinent to cancer phenotypes. Moreover, the algorithm is also capable of finding the functional relevance of uncharacterized or unexpected genes.

Comparing with the previous methods of finding driver mutation pathways, our algorithm is superior in the following two aspects. Firstly, the complexity of the solution is reduced by constructing gene networks from somatic mutation data. Secondly, there is no need to assign the number of genes in a driver pathway with our algorithm. It is necessary to point out that our algorithm does not use gene interaction data, pathways and other biological information. The algorithm could provide a supplement to the analyses of cancer data and does not replace human analyses. We also anticipate that this method will be increasingly helpful in producing hypotheses that will drive some specific experiments and increase understanding for cancer progression. Further development is anticipated with the use of machine learning techniques [28]. We also plan to analyze temporal gene mutation data with the use of spiking neural networks using the method suggested in [29,30].

## List of abbreviations used

NBM: Network-Based Method; Dendrix: *De novo *Driver Exclusivity; RME: Recurrent and Mutually Exclusive; TCGA: The Cancer Genome Atlas; KEGG: Kyoto Encyclopedia of Genes and Genomes; HNSCC: Head and Neck Squamous Cell Carcinoma Data; GBM: Glioblastoma Multiforme Data; LC: Lung Carcinoma Data; OC: Ovarian Carcinoma Data.

## Competing interests

The authors declare that they have no competing interests.

## Authors' contributions

Conceive and design the experiments: HW LG. Perform the experiments: HW XY. Analyze the data: HW FL. Contribute reagents/materials/analysis tools: HW FS. Write the paper: HW. Consult on the final version of the paper and edit the paper: HW LG NK. The authors read and approve the final version of the manuscript.

## Declarations

The publication of this article is supported by the China Scholarship Council, NSFC (Grant No. 91130006, 61202175, 61432010, 61402349, 61174162) and the Fundamental Research Funds for the Central Universities (Grant No. BDZ021404). The funders had no role in study design, data collection and analysis, decision to publish, or preparation of the manuscript.

This article has been published as part of *BMC Bioinformatics *Volume 16 Supplement 5, 2015: Selected articles from the 10th International Symposium on Bioinformatics Research and Applications (ISBRA-14): Bioinformatics. The full contents of the supplement are available online at http://www.biomedcentral.com/bmcbioinformatics/supplements/16/S5.
